# Supplementary material for: Dynamic coding and sequential integration of multiple reward attributes by primate amygdala neurons
Source: Nat Commun. 2025 Apr 1;16:3119. doi: 10.1038/s41467-025-58270-y (PMC11962072; doi:10.1038/s41467-025-58270-y)
Supplement: Supplementary file 1 — Supplementary Information [file 41467_2025_58270_MOESM1_ESM.pdf]

SUPPLEMENTARY INFORMATION

Dynamic coding and sequential integration of multiple reward attributes by primate amygdala neurons

Fabian Grabenhorst and Raymundo Báez-Mendoza

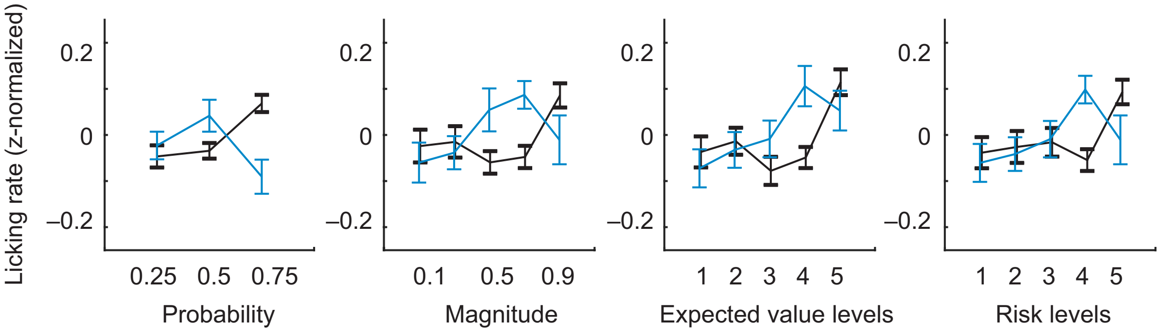

Fig. S1. Licking data. Lick rates as a function of reward probability, magnitude, expected value and risk. Blue/black: animal A/B. (See Table S1 for details).

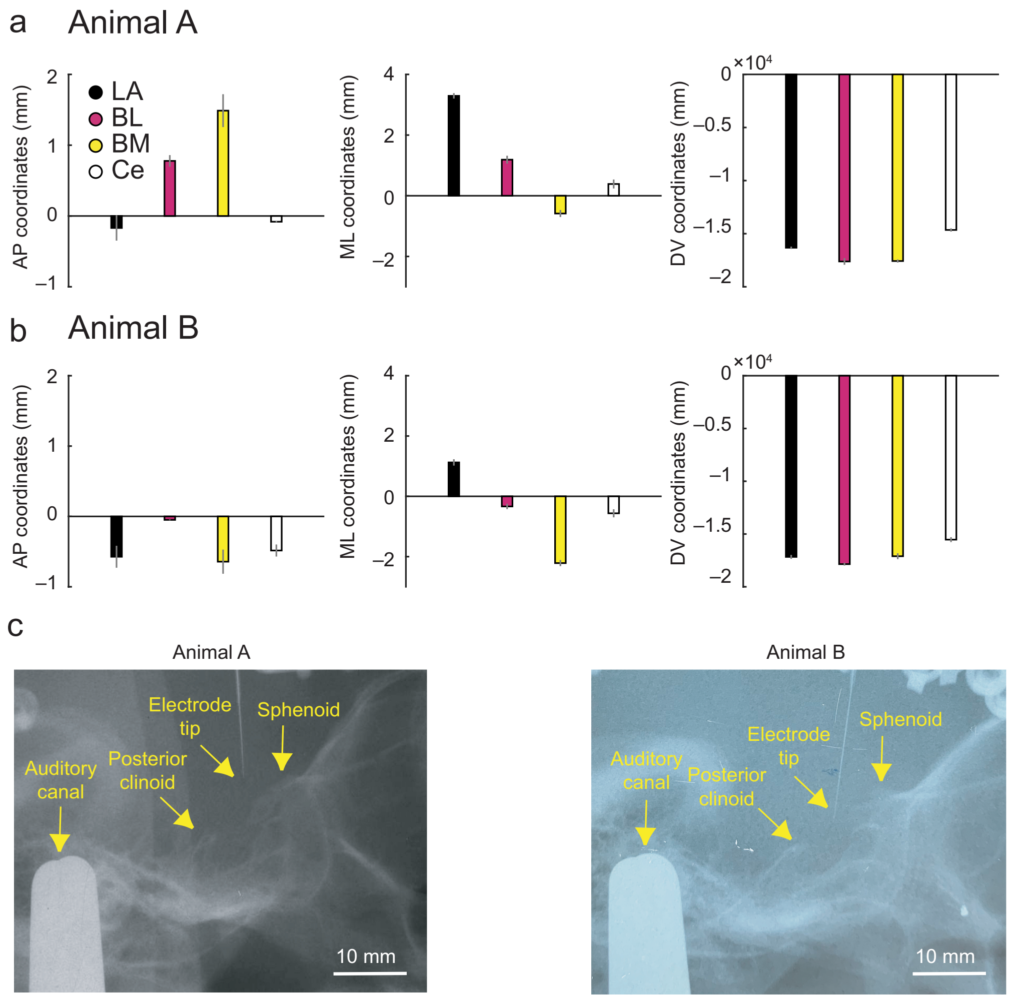

d

		Nucleus			
	**Total**	**LA**	**BL**	**BM**	**Ce**
Animal A	176	48	82	14	32
Animal B	307	65	90	41	111
Both	483	113	172	55	143

Fig. S2. Overview of recording sites in amygdala. (a, b) Mean coordinates of recording positions (± s.e.m) in anterior-posterior (AP), medio-lateral (ML) and dorsoventral (DV) dimensions for animals A and B. Coordinates shown separately for different amygdala nuclei as indicated by the color code. Coordinates are referenced to recording grid system. Recording positions were determined using histological reconstructions based on electrolytic lesions and marker pins, and stereotaxically referenced coordinates for recordings for each neuron. Reconstructed recording positions were overlaid onto a stereotaxic atlas[1] of the macaque brain at different anterior-posterior levels and neurons were assigned to different amygdala nuclei. **(c)** Lateral-view X-rays for animals A and B indicate the position of the recording electrode relative to bone landmarks and auditory canal; amygdala is located posterior and ventral to the anterior sphenoid bone and anterior to the posterior clinoid process. **(d)** Recorded amygdala neurons in different nuclei and in both animals

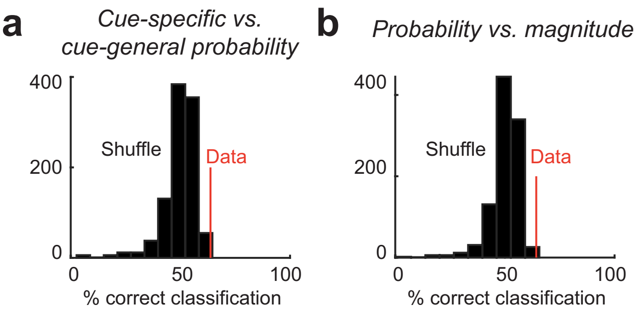

Fig. S3. Robustness of neuronal classification based on angle of regression coefficients. (a) 113 neurons coding cue-specific or cue-general probability (Fig. 2) (b) 140 neurons coding probability and/or magnitude (related to Figs. 2c and 3). We tested whether the probability regression coefficients shown in Fig 2c provided evidence for clustering into two groups corresponding to cue-general and cue-specific probability-coding neurons. We performed nearest-neighbor classification on the angles in the space of value coefficients. We were primarily interested in whether, for a given neuron, this angle would fall onto either of the two axes corresponding to cue-specific probability coding or whether the angle would lie on the diagonal between these axes, corresponding to cue-general probability coding. We therefore sign-corrected the coefficients (i.e., ignoring whether a given neuron would code probability for fractal or sector stimuli) resulting in angles between 0 and 45 degrees. For each probability-coding neuron (N = 148), we calculated the Euclidean Distance between its probability coefficients and those of the remaining neurons (leave-one-out cross-validation) to classify the tested neuron as showing either cue-specific or cue-general probability coding, depending to which group the Euclidean Distance was smallest. Classification based on the closest 'two-groups neighbor' (the mean coefficients from cue-specific and cue-general coding) resulted in 86 correct classifications (76% correct; compared to 8.4% correct based on shuffled data). **(b)** Similarly, for each probability- or magnitude-coding neuron (N = 140), we calculated the Euclidean Distance between its coefficients and those of the remaining neurons (leave-one-out cross-validation) to classify the tested neuron as coding either (i) probability or magnitude or (ii) probability and magnitude, depending to which group the Euclidean Distance was smallest. Classification based on the closest 'two-groups neighbor' (the mean coefficients from object-value and view-based value neurons) resulted in 90 correct classifications (64% correct; compared to 7.9% correct based on shuffled data).

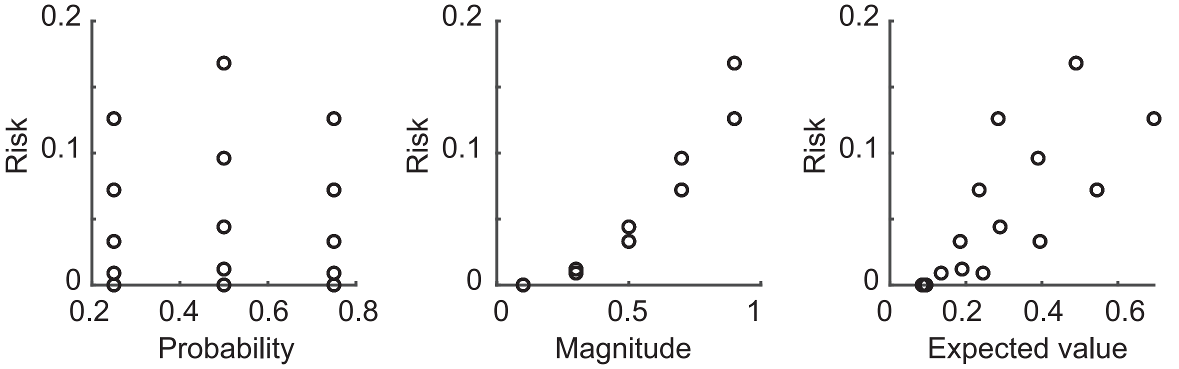

Fig. S4. Relationship of risk to probability, magnitude and expected value. Variables are plotted for a typical testing session.

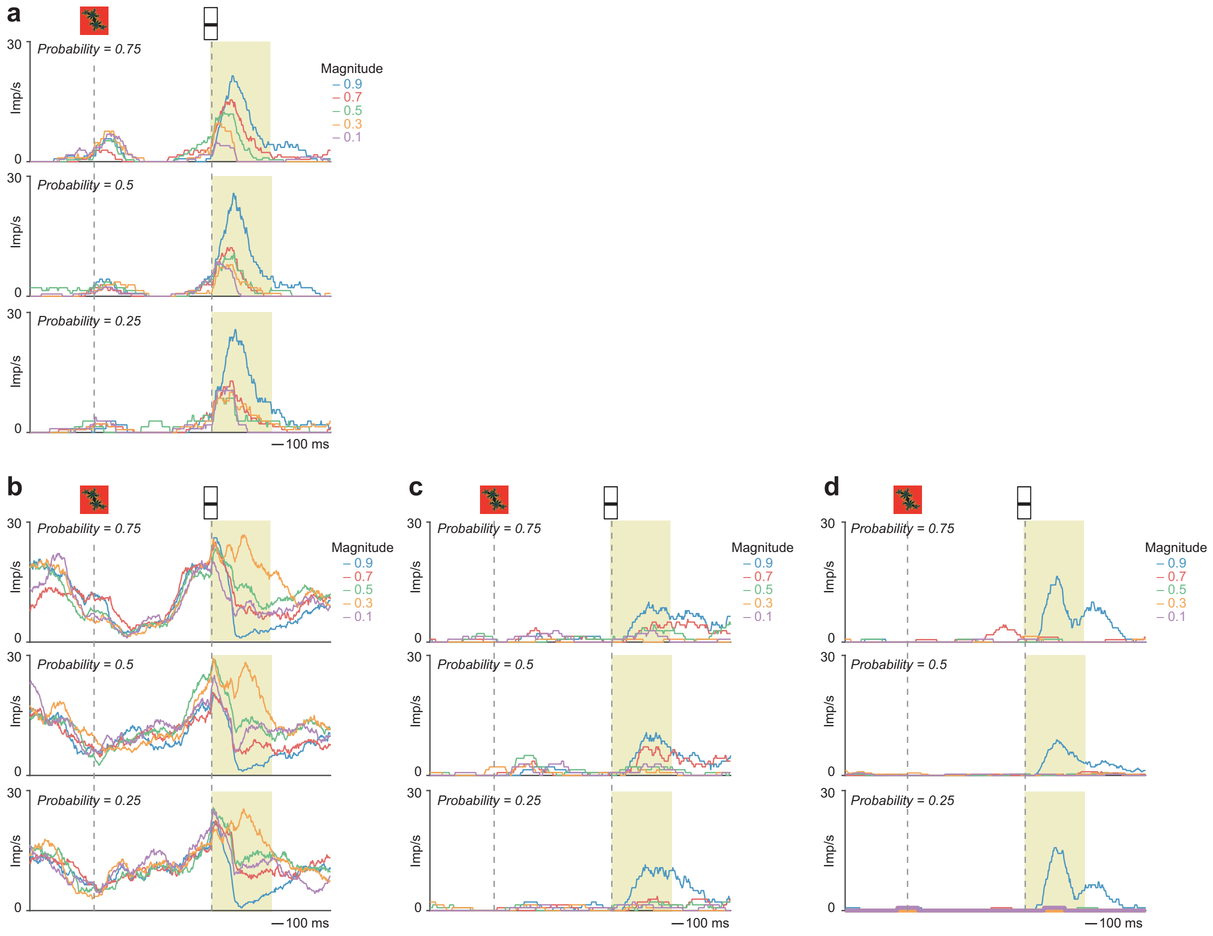

Fig. S5. Amygdala neuron coding risk. (a) Activity of the neuron in Fig. 4a, shown separately for different probability and magnitude levels. **(b-d)** Activity of the neurons in Fig. 4i-k, shown separately for different probability and magnitude levels.

a

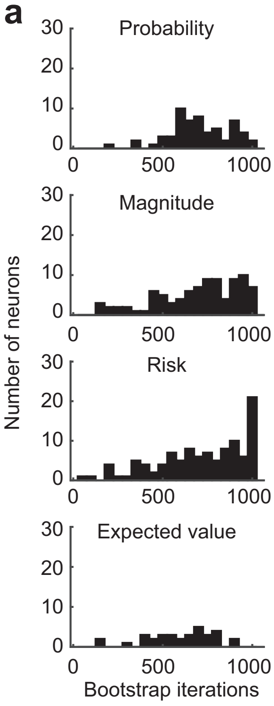

b

Risk neurons (N = 99, eq. 4)			
		Magnitude	
Risk		*nsig*	*sig*
	nsig	4	0
	sig	38	57

Probability neurons (N = 61, eq. 4)			
		Risk	
Probability		*nsig*	*sig*
	nsig	5	0
	sig	40	16

Magnitude neurons (N = 94, eq. 4)			
		Risk	
Magnitude		*nsig*	*sig*
	nsig	6	7
	sig	25	56

Probability neurons (N = 61, eq. 4)			
		Magnitude	
Probability		*ns*	*sig*
	nsig	5	0
	sig	27	29

c

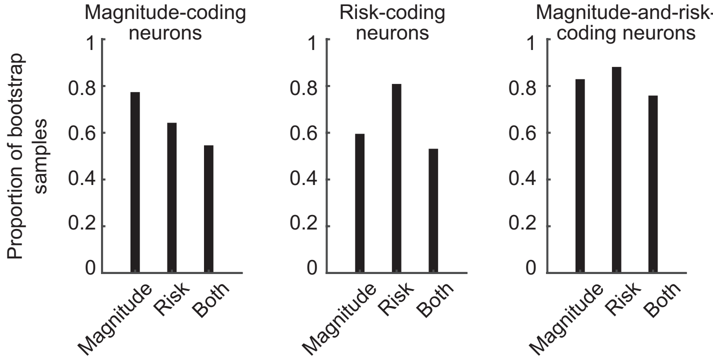

Fig. S6. Robustness of neuronal classification based on bootstrap. (a) Distribution of neurons with significant regressor in sliding-window regression (eq. 4, GLM4) across 1,000 iterations from a bootstrap analysis (1,000 iterations per neuron, with resampling). Probability, magnitude and risk were more frequently confirmed in bootstrap (number of significant bootstrap iterations) than expected value, which was encoded by the lowest number of neurons in the original analysis. Further, a minority of neurons encoding the correlated variables magnitude and risk showed less reliable coding, indicated by relatively lower number of significant iterations. Thus, in these neurons, the distinction between magnitude and risk coding was likely less clear; see results in (b). (b) Confusion matrices for key variables. Each matrix considers neurons that coded a particular variable in our main model (eq. 4) and specifies how many of these neurons were identified in the bootstrap as coding this variable but not a control variable (row/column: 'sig'/'nsig'; sig: significant, nsig: non-significant), neurons that in the bootstrap coded the control variable only but not the originally coded variable ('nsig'/'sig'), and neurons that in the bootstrap coded both ('sig'/'sig') or neither variables ('nsig'/'nsig'). Significance defined as a significant regressor in more than 500 iterations of the bootstrap. For example, of 99 risk-coding neurons in the original analysis (upper left matrix in (b)), 38 coded risk but not magnitude in the bootstrap, 57 coded both risk and magnitude, 0 coded magnitude but not risk, and 4 coded neither variable. (c) Proportion of bootstrap samples classified as magnitude-coding, risk-coding or joint magnitude-and-risk-coding shown separately for neurons that were in the main analysis classified as magnitude-coding (left), risk-coding (middle), or joint magnitude-and-risk-coding (right).

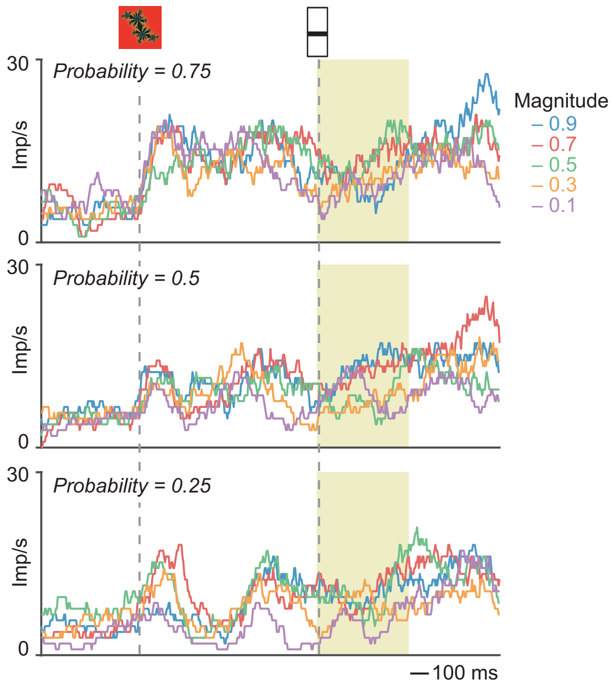

Fig. S7. Amygdala neuron coding expected value. Activity of the neuron in Fig. 5a, shown separately for different probability and magnitude levels.

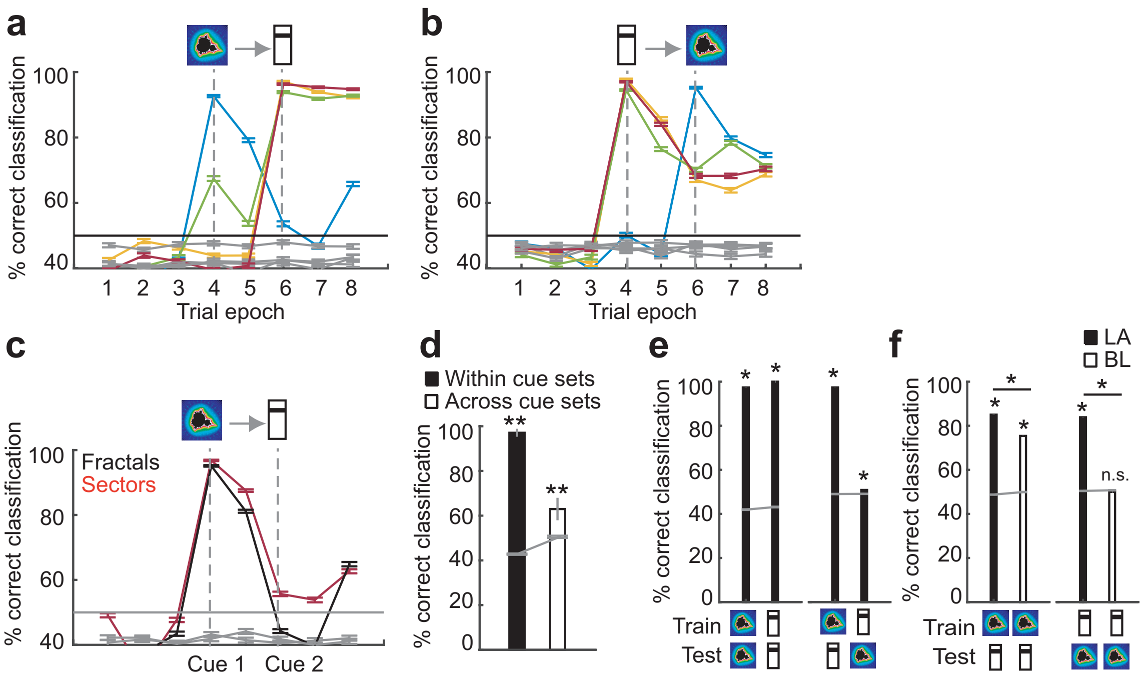

Fig. S8. Population decoding using support-vector machine algorithm. (a) Accuracy of a support-vector machine decoder (mean +/- s.e.m.) classifying high-vs.-low levels of probability, magnitude, expected value (EV), and risk from unselected amygdala neurons (N = 483) in specific trial epochs. Gray lines: decoding based on data using trial-shuffled group labels. **(b)** Decoding accuracy in control task with reversed cue order. **(c)** Decoding accuracy for probability based on fractal or sector cue trials. Gray lines: decoding based on data using trial-shuffled group labels. **(d)** Decoding accuracy (mean +/- s.e.m.) for probability when training and testing within cue set (fractal or sector, black bar) and training and testing across cue sets (averaged over fractal-to-sector and sector-to-fractal decoding, white bar). Gray lines: decoding based on data using trial-shuffled group labels. **(e)** Decoding and cross-decoding of probability and magnitude information. Decoders were trained to discriminate low-vs.-high probability (first cue period, indicated by fractal symbol, though the analysis included both fractal and sector trials) or magnitude (second cue period, indicated by bar symbol) and tested within or across periods and reward variables. Gray lines: decoding based on data using trial-shuffled group labels. **(f)** Decoding and cross-decoding performed separately for LA (N = 113) and BL (N = 172) neurons. Gray lines: decoding based on data using trial-shuffled group labels. *: $P < 0.001$; n.s.: non-significant. Statistical significance assessed with two-sided t-test (no adjustment for multiple comparisons).

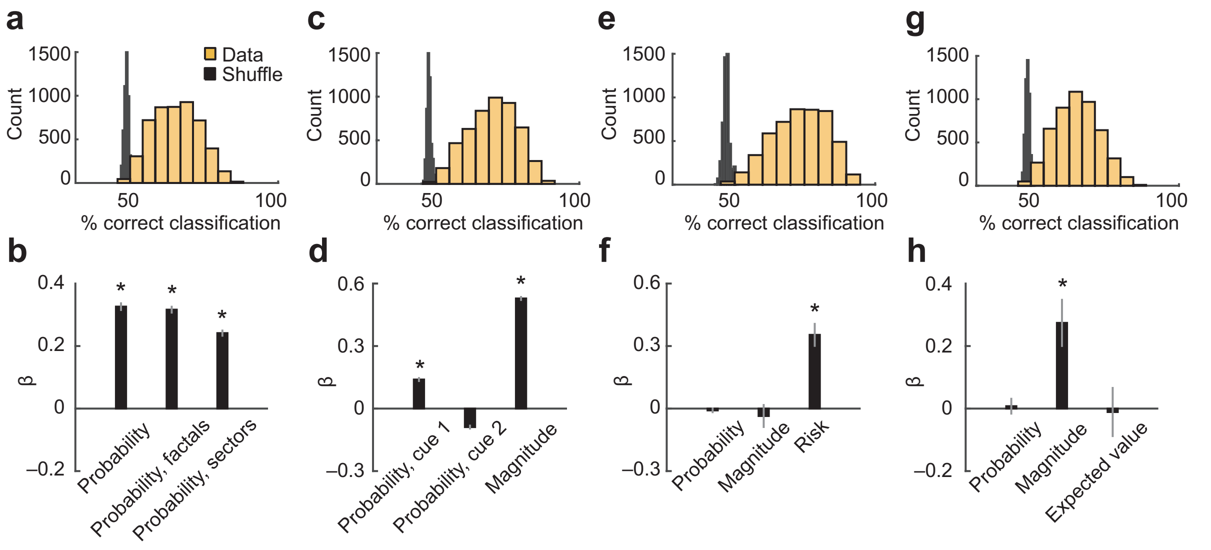

Fig. S9. Population decoding from randomly selected neuronal subsets. (**a**) Distribution of probability-decoding accuracy for 5,000 randomly selected small (N = 20) subsets of amygdala neurons. **(b)** Regression of probability-decoding accuracy on sample mean of single-neuron probability betas. **(c)** Distribution of magnitude-decoding accuracy for 5,000 randomly selected small (N = 20) subsets of amygdala neurons. **(d)** Regression of magnitude-decoding accuracy on sample mean of single-neuron probability (cue 1 and cue 2) and magnitude betas. **(e)** Distribution of risk-decoding accuracy for 5,000 randomly selected small (N = 20) subsets of amygdala neurons. **(f)** Regression of risk-decoding accuracy on sample mean of single-neuron probability, magnitude and betas. **(g)** Distribution of expected value-decoding accuracy for 5,000 randomly selected small (N = 20) subsets of amygdala neurons. **(h)** Regression of expected value-decoding accuracy on sample mean of single-neuron probability, magnitude and expected-value betas. Statistical significance assessed with two-sided t-test (no adjustment for multiple comparisons).

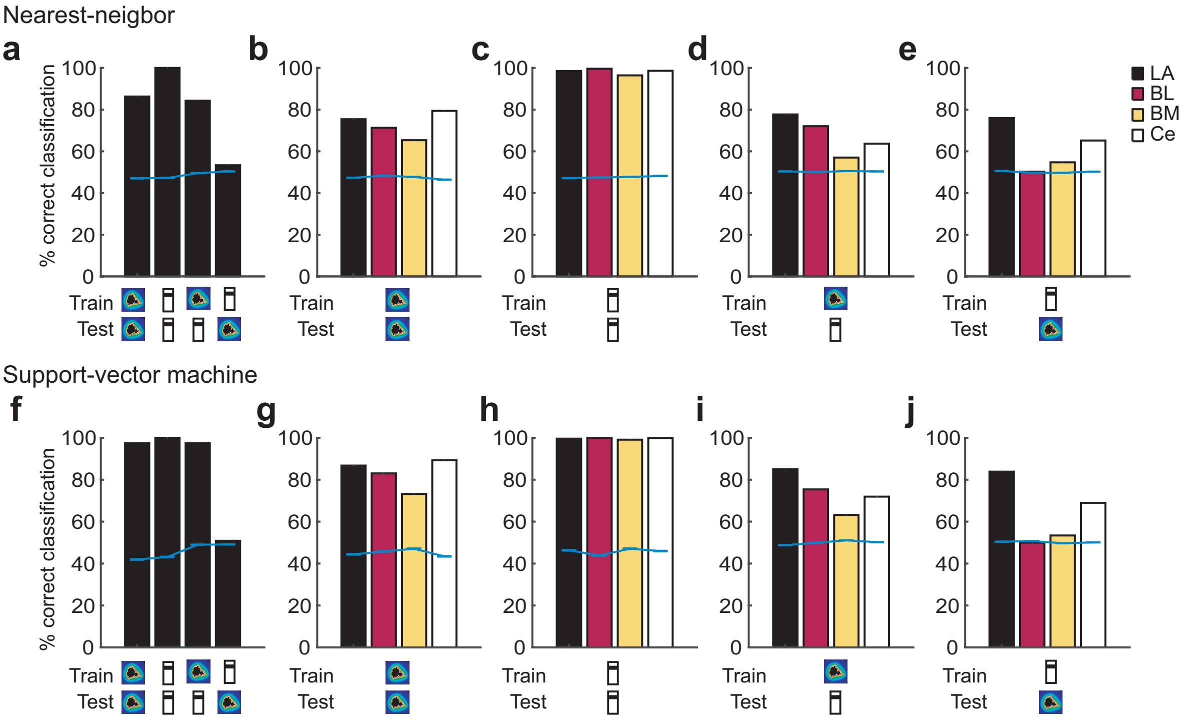

Fig. S10. Decoding and cross-decoding of probability and magnitude information using nearest-neighbor and support-vector machine classifiers. Classifiers were trained to discriminate low-vs.-high probability (first cue period, indicated by fractal symbol, though the analysis included both fractal and sector trials) or magnitude (second cue period, indicated by bar symbol) and tested within or across periods and reward variables. (a) and (f) show decoding results from all recorded amygdala neurons; panels show decoding for different amygdala nuclei. (b, g) Training and testing on probability; (c, h) training and testing on magnitude; (d, i) training on probability and testing on magnitude; (e, j) training on magnitude and testing on probability.

Table S1. Mixed-effects regression of licking behavior on task variables. (Statistical significance assessed with two-sided t-test (no adjustment for multiple comparisons).)

**Variable**	**Estimate**	**Standard error**	**t-statistic**	**Degrees of Freedom**	**P-value**
**Animal A**					
Intercept	-0.266	0.028	-9.379	12,245	7.7e-21
Probability	0.188	0.043	4.314	12,245	1.6e-5
Magnitude	0.350	0.131	11.129	12,245	1.2e-28
**Animal B**					
Intercept	-0.169	0.037	-4.474	6,473	7.7e-6
Probability	0.176	0.059	2.940	6,473	0.003
Magnitude	0.170	0.042	3.991	6,473	6.6e-5

Table S2. Correlations between regressors in GLM 4 (Pearson correlation coefficients).

	Probability	Magnitude	Expected value	Risk
Probability	1			
Magnitude	-0.003	1		
Expected value	0.486	0.806	1	
Risk	-0.003	0.943	0.760	1

References

1. Paxinos, G., Huang, X.-F. & Toga, A.W. *The rhesus monkey brain in stereotaxic coordinates* (Academic Press, San Diego, CA, 2000).
